# One Small Step for a Man: Estimation of Gender, Age and Height from Recordings of One Step by a Single Inertial Sensor

**DOI:** 10.3390/s151229907

**Published:** 2015-12-19

**Authors:** Qaiser Riaz, Anna Vögele, Björn Krüger, Andreas Weber

**Affiliations:** 1 Department of Computer Science II, Universität Bonn, Bonn 53113, Germany; voegele@cs.uni-bonn.de (A.V.); weber@cs.uni-bonn.de (A.W.); 2 Gokhale Method Institute, Stanford, CA 94305, USA; kruegerb@cs.uni-bonn.de

**Keywords:** estimation of soft biometrics, gender, age and height estimation from inertial data, gait analysis, inertial sensors to estimate gender, age and height, accelerometers

## Abstract

A number of previous works have shown that information about a subject is encoded in sparse kinematic information, such as the one revealed by so-called point light walkers. With the work at hand, we extend these results to classifications of soft biometrics from inertial sensor recordings at a single body location from a single step. We recorded accelerations and angular velocities of 26 subjects using integrated measurement units (IMUs) attached at four locations (chest, lower back, right wrist and left ankle) when performing standardized gait tasks. The collected data were segmented into individual walking steps. We trained random forest classifiers in order to estimate soft biometrics (gender, age and height). We applied two different validation methods to the process, 10-fold cross-validation and subject-wise cross-validation. For all three classification tasks, we achieve high accuracy values for all four sensor locations. From these results, we can conclude that the data of a single walking step (6D: accelerations and angular velocities) allow for a robust estimation of the gender, height and age of a person.

## 1. Introduction

Sparse representation of human motions has been investigated for some decades now. It is well-known that representation of human motion by point light displays and similar concepts (e.g., point light walker [1,2]) contains detailed information on several aspects of motions and their initiators.

Over the years, the possibilities to identify certain parameters characterizing given motions have been explored. On the one hand, it is possible to discover information about the displayed motions as such. In the field of action recognition, it has been shown that estimation of poses and skeletons from video and motion capture data allows for recognition and analysis of human movement (Lv *et al.* [3], Junejo *et al.* [4], Barnachon *et al.* [5], Oshin *et al.* [6]). The survey of vision-based human motion capture by Moeslund *et al.* [7] discusses the advances and application of motion-capture-related techniques for tracking, pose estimation and recognition of movement. Recognition of motion patterns from video data can be achieved by machine learning approaches exploiting local space-time features (e.g., for SVM-based methods, Schüldt *et al.* [8]). On the other hand, information on the kinematic properties of living beings or animated objects can be detected by analyzing representations of motions. This can be done using motion capture data from passive or active devices, as well as contact forces measurements (Venture *et al.* [9], Kirk *et al.* [10]).

More recently, the market for wearable devices has virtually exploded (Liew *et al.* [11], Son *et al.* [12]). The sheer number of devices [13] reflects that there are numerous methods to capture and analyze human motion in a relatively new field of application associated with ubiquitous computing. Even though information acquired by such devices may be less accurate than information acquired by modern motion capture systems (Le Masurier *et al.* [14], Foster *et al.* [15]), it has been shown that reconstruction of motion from extremely sparse sensor setups is possible in practice (Tautges *et al.* [16], Riaz *et al.* [17]). This indicates that data collected using tri-axial accelerometers are suitable for classification tasks, e.g., associated with social actions (Hung *et al.* [18]), general everyday activities (Parkka *et al.* [19], Jean-Baptiste *et al.* [20], Dijkstra *et al.* [21]) or repetitive physical exercises (Morris *et al.* [22]).

We investigated if data from a single wearable sensor can reveal similar information about the moving subject as motion capture data in the sense of the above-quoted [1,2]. We focus on classification of gender, age and height defining exemplary inertial properties of moving subjects. Our experiments show that it is indeed possible to classify and thereby estimate such properties. Our method is able to process representations of single steps recorded by one accelerometer (as opposed to longer data sequences; Neugebauer *et al.* [23]). In sum, our method is able to recover soft biometric information with high accuracy consistently over various sensor positions. Since the classification task depends on the chosen feature sets, we further investigated this by evaluating the role of different possible feature sets in the classification.

Modern machine learning techniques like decision trees can target pattern recognition and prediction tasks based on many different representations of motion (Brand *et al.* [24], Bao *et al.* [25], Kwapisz *et al.* [26]). We used random forests, a learning method based on the construction of multiple decision trees, which can be used for classification, as well as regression tasks. While learning predictive models by using decision trees on their own may result in over-fitting to a training set (Phan *et al.* [27]), random forests are less prone to this problem. For an overview of random forests, refer to the works of Breimann [28] or Liaw and Wiener [29].

## 2. Materials and Methods

### 2.1. Participants’ Consent

All participants were informed in detail about the purpose of the study, the nature of the experiments, the types of data to be recorded and the data privacy policy. The subjects were aware that they were taking part in experiments where a number of biometric and kinematic properties were monitored. The main focus of the study was communicated to the subjects during their progress over the course of the training by the specialists of Gokhale Method Institute [30] (Stanford, CA, United States). Each willing participant was asked to fill in the data collection form with personal details, including full name, sex, age and height.

### 2.2. Population Characteristics and Sampling

The participants were selected during a gait and posture training program conducted in July of 2014 by the specialists of Gokhale Method Institute. They use special gait and posture training methods to help regain the structural integrity of the body. The training program consisted of six 90-minute training sessions. The study population consisted of a total of 26 adults with a male to female ratio of 12:14 and an average age of 48.1 years (*σ* = ± 12.7). The average height of the participants was recorded at 174 cm (*σ* = ± 10.2). The characteristics of the study population are shown in Table 1.

**Table 1:** Characteristics of the study population, including age, sex and height. For validation, two types of models were used: k-fold cross-validation and subject-wise cross-validation.

Variable	Characteristics
**Total Population**	26
**Age** (y, mean, ± SD)	48.1 ± 12.7
**Female Participants**	14
**Male Participants**	12
**Height** (cm, ± SD)	174 ± 10.2

A *k*-fold cross-validation model (chosen value of *k* = 10) was used to compute the classification accuracy of the classifier. In *k*-fold cross-validation, original sample data are randomly partitioned into *k* equally-sized sub-samples or folds. Out of the *k* folds, *k*-1folds are used for training, and the left-out fold is used for validation. The cross-validation process is repeated *k* times, and each of the *k* folds is used exactly once for validation. For sampling, the stratified sampling method [31] is used to divide the population into training and test datasets.

A subject-wise cross-validation model was also employed to compute the classification accuracy of each participant against others. Subject-wise cross-validation is a special variant of leave-one-out cross-validation in which instead of leaving one sample out for validation, all samples of one participant are left out for validation. For *n* participants (*n* = 26, in our case), all samples of n-1 participants are used for training, and all samples of the left-out participant are used for testing. The cross-validation process is repeated *n* times in order to validate each participant exactly once against the rest. Unlike 10-fold cross-validation, the number of samples in each fold is not equal in subject-wise cross-validation. This is due to the difference in the step length of each subject. Subjects with shorter step lengths have more steps than the others.

### 2.3. Standardized Gait Tasks

The gait task consisted of a 10-meter straight walk from a starting point, turning around and walking back to the starting point. Participants were asked to walk in their natural manner and to repeat the gait task two times, resulting in a 4×10-meter walk. Three different types of experiments were performed: (1) walking on a hard surface (concrete floor) with shoes on; (2) walking on a hard surface (concrete floor) with bare feet; and (3) walking on a soft surface (exercise mattress) with bare feet. Data were recorded during three different stages of the training course: (1) at the start of the training (before the 1st session); (2) in the middle of the training (after the 3rd session); and (3) at the end of the training (after the 6th session). Hence, for each participant, 9 different recording sessions were carried out in total (see Table 2).

**Table 2:** Standardized gait tasks. Experiments were performed on different surfaces with and without shoes, as shown here. For each participant, 9 different recording sessions were carried out in total.

		4 × 10-Meter Straight Walk		
		Hard Surface	Hard Surface	Soft Surface
		Shoes On	Barefoot	Barefoot
**Recordings**	**Before 1st Session**	√	√	√
	**After 3rd Session**	√	√	√
	**After 6th Session**	√	√	√

### 2.4. Sensor Placement and Data Collection

**Figure 1:**
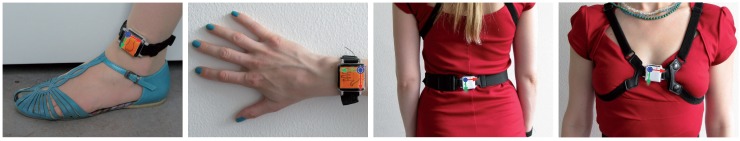
Placement of four APDM Opal IMUs on different body parts. The sensors were placed on four different locations: left ankle, right wrist, lower back and chest.

A set of four APDM Opal wireless inertial measurement units [32] was used to record accelerations and angular velocities. An APDM Opal IMU consists of a triad of three accelerometers and three gyroscopes. The technical specifications of the sensor are given in Table 3. The sensors were tightly attached to different body parts using adjustable elastic straps. We were particularly interested in the inertial measurements of four different body parts: (1) chest; (2) lower back; (3) right wrist; and (4) left ankle. The sensor placement at each body part is shown in Figure 1.

**Table 3:** Technical specifications of the APDM Opal IMU.

	Accelerometer	Gyroscope	Magnetometer
**Axes**	3 axes	3 axes	3 axes
**Range**	±2 g or ±6 g	±2000 deg/s	±6 Gauss
**Noise**	0.0012 m/s${}^{2}$/$\sqrt{Hz}$	0.05 deg/s/$\sqrt{Hz}$	0.5 mGauss/$\sqrt{Hz}$
**Sample Rate**	1280 Hz	1280 Hz	1280 Hz
**Output Rate**	20 to 128 Hz	20 to 128 Hz	20 to 128 Hz
**Bandwidth**	50 Hz	50 Hz	50 Hz
**Resolution**	14 bits	14 bits	14 bits

### 2.5. Pre-Processing

The output sampling rate of an APDM Opal IMU sensor is adjustable between 20 and 128 Hz. In our experiments, an output sampling rate of 128 Hz was chosen. Due to the noisy nature of the acceleration measurements, raw data were pre-processed to suppress noise. To this end, we used the moving average method with a window size of 9 frames to smooth the raw signal and suppress noise.

### 2.6. Signal Decomposition

**Figure 2:**
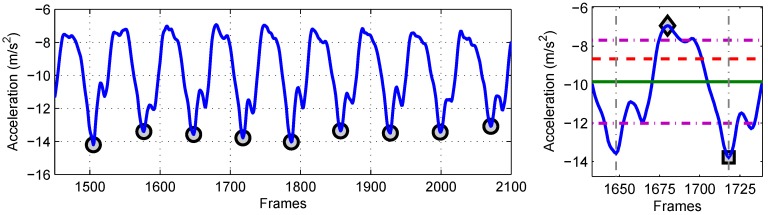
The pre-processed input signal from the x-axis of the IMU’s accelerometer attached to the lower back and an extracted single step are shown. In the left image, detected valleys are highlighted with ◯. In the right image, a decomposed signal depicting a single step is shown between the vertical dash-dot lines (- ·). Some of the extracted features from the single step are: (1) square (□): global minimum; (2) diamond (◇): global maximum; (3) solid line (–): mean; (4) horizontal dash-dot line (- ·): standard deviation; (5) dashed line (- -): root mean square; (6) between vertical dash-dot lines (- ·): length and duration.

The input signal consists of a long sequence of steps, which is segmented into single steps in order to extract features. A simple approach to decompose a long sequence of steps into single steps is by means of peak and valley detection [33,34,35]. In this approach, peaks are detected by finding local maxima, whereas valleys are detected by finding local minima. The detection of false peaks is minimized by using two thresholds: $\Delta_{d}$ and $\Delta_{h}$·$\Delta_{d}$ is used to define the minimum distance between two peaks, and $\Delta_{h}$ is used to define the minimum height of the peak. We have used the same approach to detect peaks and valleys from the input signal. The values of the two thresholds are chosen by experimentation. The valleys are then used to cut the input signal into individual steps. Peaks and valleys are only detected in the x-axis of the acceleration signal and are used to decompose the y- and z-axes of acceleration and all axes of the gyroscope. This approach makes sure that the length of the individual step is consistent in all axes of the acceleration and gyroscope. In Figure 2, the left side image presents the pre-processed input signal from the x-axis of the IMU’s accelerometer attached to the lower back. The detected valleys, highlighted with circles (◯), are also shown.

### 2.7. Extraction of Features

All single steps detected from the signal decomposition are further processed to extract different features from the time and frequency domains. Table 4 presents a complete list of features extracted from different components of accelerations and angular velocities. For each single step, the feature set consists of 50 features in total. Statistical features include: step length, step duration, average, standard deviation, global minimum, global maximum, root mean square and entropy. Energy features include the energy of the step. The maximum amplitude of the frequency spectrum of the signal is calculated using fast Fourier transform (FFT). The step length and the step duration are only computed for the x-axis of the accelerations, as they remain the same in all other axes. All of the remaining features are computed for all 3D accelerations and 3D angular velocities. In Figure 2, the right-hand image presents a decomposed signal depicting a single step between the vertical dash-dot lines (- ·). Some of the extracted features are also shown, including: (1) square (□): global minimum; (2) diamond (◇): global maximum; (3) solid line (–): mean; (4) horizontal dash-dot line (- ·): standard deviation; (5) dashed line (- -): root mean square; (6) between vertical dash-dot lines (- ·): length and duration of the step.

**Table 4:** Description of the extracted features for each step from the accelerometer (A) and/or the gyroscope (G). For each step, 50 features from the time and frequency domains are computed.

Feature Name	Sensor	Axis	Total	Description
**Step Length**	A	x	1	Total number of frames
**Step Duration (s)**	A	x	1	Step duration in seconds
**Average**	A, G	x, y, z	6	Mean value of the step
**Standard Deviation**	A, G	x, y, z	6	*σ* of the step
**Minimum**	A, G	x, y, z	6	Global minimum of the step
**Maximum**	A, G	x, y, z	6	Global maximum of the step
**Root Mean Square**	A, G	x, y, z	6	RMS value of the step
**Entropy**	A, G	x, y, z	6	Uncertainty measure of the step, $s_{i}$.: $-\sum_{i=1}^{n}\left(p_{i}\right)log_{2}\left(p_{i}\right)$ where $p_{i}=\frac{\frac{s_{i}}{max(s_{i})}}{\sum_{i=1}^{n}\frac{s_{i}}{max(s_{i})}}$
**Signal Energy**	A, G	x, y, z	6	Energy of the step: $\sum_{n=1}^{N}{\left|x\left[n\right]\right|}^{2}$
**Amplitude**	A, G	x, y, z	6	Maximum amplitude of the frequency spectrum of the signal of the step

### 2.8. Classification of Features

Training and validation data were prepared for each sensor using the features extracted in the previous step. Three types of group classification tasks were performed: (1) gender classification; (2) height classification; and (3) age classification. Furthermore, training and validation data were also prepared for classification within participant subgroups for height and age classification. In Table 5, the characteristics of the population within different classification tasks are presented. For age and height classification, we choose classes based on the available data. Here, we have tried to define meaningful thresholds for classes while keeping balanced populations for all classes.

**Table 5:** Characteristics of the population within different group and subgroup classification tasks.

Task	Classes	N	Age (Mean ± SD)
**Group Classification Tasks**			
**Gender Classification**	Male	12	43.75 ± 14.50
	Female	14	51.79 ± 11.15
**Age Classification**	Age <40	9	34.11 ± 03.62
	40< Age <50	6	46.67 ± 02.58
	Age ≥50	11	60.67 ± 07.48
**Height Classification**	Height ≤ 170 cm	8	55.62 ± 11.29
	170 cm < Height < 180 cm	10	44.70 ± 11.31
	Height ≥180 cm	8	44.75 ± 13.81
**Subgroup Classification Tasks**			
**Age Classification**			
**Male Group**	Age ≤40	6	32.67 ± 02.94
	Age >40	6	54.83 ± 09.87
**Female Group**	Age ≤50	6	41.83 ± 06.08
	Age >50	8	59.25 ± 07.48
**Height Classification**			
**Male Group**	Height ≤180 cm	7	38.43 ± 11.27
	Height >180 cm	5	51.20 ± 13.85
**Female Group**	Height ≤ 170 cm	8	55.62 ± 11.29
	Height >170 cm	6	46.67 ± 09.48

As the classifier, random forest [29] was chosen and trained on the training dataset with the following values of parameters: number of trees = 400; maximum number of features for best split = 7. Two types of validation strategies were employed: stratified 10-fold cross-validation and subject-wise cross-validation. The 10-fold cross-validation was employed for all group and subgroup classification tasks, whereas the subject-wise cross-validation was employed to group classification tasks only.

For each sensor in a classification task, the classifier was trained and validated for three different sets of features: (1) 3D accelerations (26 features); (2) 3D angular velocities (26 features); and (3) 6D accelerations and angular velocities (50 features). The 10-fold cross-validation was employed for all three sets of features, whereas the subject-wise cross-validation was employed for the third set of features (50 features) only. Finally, the classification rate, specificity, sensitivity and the positive predictive value (PPV) for each set of features were calculated as explained in [36]. The same approach was used for all group and subgroup classification tasks. The classification rate *c* or classification accuracy is given by the formula in Equation (1):

(1) $$c=\frac{\left(TP+TN\right)}{\left(TP+TN+FP+FN\right)}$$

where TP, TN are the numbers of true positives and true negatives, respectively, and FP, FN are the numbers of false positives and false negatives, respectively.

## 3. Results

In the following sections, we present the results of our investigations of the recorded gait data. Our classification results prove a number of hypotheses regarding biometric and biographic characteristics of the human subjects. Specifically, the gender, the body height and the age of participants could be classified well. Each of classification tasks was solved by training random forest classifiers, as introduced in the previous section.

### 3.1. Gender Classification

Our goal was to show that classification tasks regarding the gender of the trial subject can be performed sufficiently well by using the proposed sensors attached to each of the given locations.

$H_{0}$: The gender can be identified by motion recordings of any of the employed sensors

**Figure 3:**
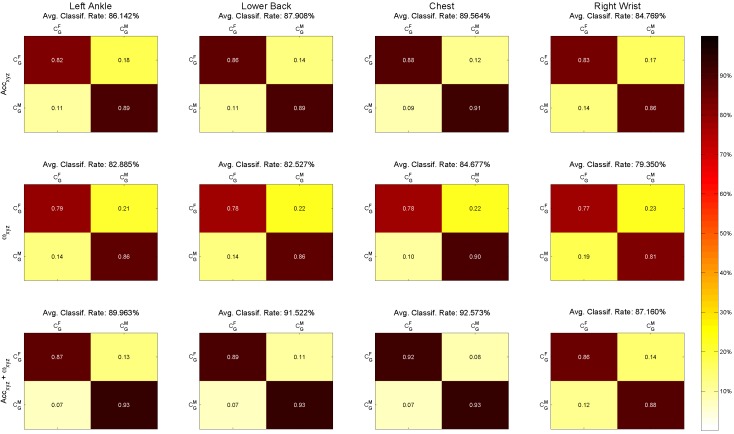
Confusion matrices of gender classification computed with 10-fold cross-validation. Each column presents sensor position (left to right): left ankle, lower back, chest and right wrist. Each row presents feature sets used for classification (top to bottom): 3D accelerations (26 features), 3D angular velocities (26 features) and 6D accelerations and angular velocities (50 features). Classes: $C_{G}^{F}$ = gender female; $C_{G}^{M}$ = gender male.

The results presented in Figure 3 show that the statement holds true for each of the four sensors individually. For each sensor, there are three different images visualizing the results of the binary classification, namely for the investigation of accelerations, of angular velocities, as well as of both combined. The confusion matrices encode the following information: each column represents the instances in one of the predicted classes, while each row represents the instances in the actual class (female/male).

For the application of acceleration only, the classification rates are higher than 84.8% for each of the sensors. Classification results based on angular velocities show a lower classification rate, but still above 79.35%. The classification based on the combined features performs better than each of the individual feature sets, namely above 87%. More precisely, the results for the combined features are (listed by sensor in descending order of rates): chest (92.57%), lower back (91.52%), left ankle (89.96%), right wrist (87.16%). Table 6 presents 10-fold cross-validation results of gender classification, including correct classification accuracy, sensitivity, specificity, the positive predictive value (PPV) of each class and the average PPV of all classes. PPV${}_{C_{1}}$ represents the PPV of the class $C_{G}^{F}$, and PPV${}_{C_{2}}$ represents the PPV of the class $C_{G}^{M}$.

**Table 6:** Classification results obtained by using 10-fold cross-validation for different classification categories: gender, height and age. The results show balanced correct classification rates, sensitivity, specificity, the positive predictive value (PPV) of each class and the average PPV of all classes.

Classification Task	Body Part	Sensor	Class.Rate	Sens.	Spec.	PPV${}_{{\text{C}}_{1}}$	PPV${}_{{\text{C}}_{2}}$	PPV${}_{{\text{C}}_{3}}$	Avg.PPV
**Gender Classification**	Chest	A${}_{xyz}$, G${}_{xyz}$	92.57	91.72	93.24	91.43	93.48	–	92.45
	Lower Back	A${}_{xyz}$, G${}_{xyz}$	91.52	89.42	93.18	91.22	91.75	–	91.49
	Right Wrist	A${}_{xyz}$, G${}_{xyz}$	87.16	85.75	88.32	85.85	88.24	–	87.05
	Left Ankle	A${}_{xyz}$, G${}_{xyz}$	89.96	86.77	92.57	90.52	89.54	–	90.03
**Body Height Classification**	Chest	A${}_{xyz}$, G${}_{xyz}$	89.05	88.84	94.45	89.65	87.43	90.00	89.03
	Lower Back	A${}_{xyz}$, G${}_{xyz}$	88.45	88.16	94.05	91.36	88.73	86.39	88.82
	Right Wrist	A${}_{xyz}$, G${}_{xyz}$	84.78	84.65	92.33	83.40	85.21	85.43	84.68
	Left Ankle	A${}_{xyz}$, G${}_{xyz}$	87.28	87.07	93.47	89.87	89.06	84.23	87.72
**Age Classification**	Chest	A${}_{xyz}$, G${}_{xyz}$	88.82	87.40	94.05	90.10	93.02	85.81	89.64
	Lower Back	A${}_{xyz}$, G${}_{xyz}$	88.82	87.20	94.12	87.34	89.48	90.03	88.95
	Right Wrist	A${}_{xyz}$, G${}_{xyz}$	83.50	81.08	91.18	82.23	88.74	82.72	84.56
	Left Ankle	A${}_{xyz}$, G${}_{xyz}$	85.74	83.80	92.33	86.09	92.52	82.82	87.14

### 3.2. Body Height Classification

Another goal was body height classification from only accelerations, angular velocities and a combination of both.

$H_{1}$: The body height can be identified by motion recordings of any of the employed sensors

**Figure 4:**
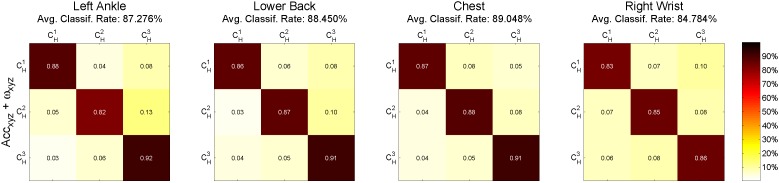
Confusion matrices of body height classification computed with 10-fold cross-validation. Each column presents the sensor position (left to right): left ankle, lower back, chest and right wrist. 6D accelerations and angular velocities (50 features) were used for classification. $C_{H}^{1}:$ height ≤170 cm, $C_{H}^{2}:$ 170 cm < height < 180 cm, $C_{H}^{3}:$ height ≥180 cm.

The results of the ternary classification for each individual sensor are given in Figure 4. Here, the classification estimated the assignment to three classes ($C_{H}^{1}:$ height ≤170 cm, $C_{H}^{2}:$ 170 cm < height < 180 cm, $C_{H}^{3}:$ height ≥180 cm). A behavior similar to the gender classification was observed where the classification based on the combined features of accelerations and angular velocities performs better than the individual ones. More precisely, the results for the combined features are (listed by sensor in descending order of rates): chest (89.05%), lower back (88.45%), left ankle (87.27%), right wrist (84.78%). Table 6 presents 10-fold cross-validation results of body height classification, including correct classification accuracy, sensitivity, specificity, the positive predictive value (PPV) of each class and the average PPV of all classes. PPV${}_{C_{1}}$ shows the PPV of the class $C_{H}^{1}$; PPV${}_{C_{2}}$ shows the PPV of the class $C_{H}^{2}$; and PPV${}_{C_{3}}$ shows the PPV of the class $C_{H}^{3}$.

### 3.3. Age Classification

Another goal was age group classification from only accelerations, angular velocities and their combination.

$H_{2}$: The age group of individuals can be identified by motion recordings of any of the employed sensors.

**Figure 5:**
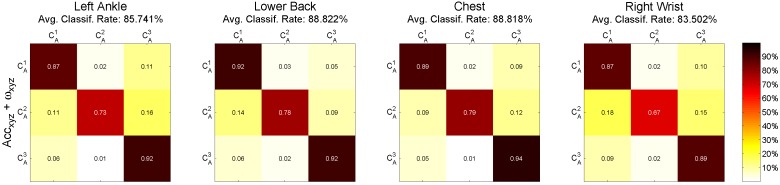
Confusion matrices of age classification computed with 10-fold cross-validation. Each column presents the sensor position (left to right): left ankle, lower back, chest and right wrist. 6D accelerations and angular velocities (50 features) were used for classification. $C_{A}^{1}$: age <40; $C_{A}^{2}$: 40 ≤ age < 50; $C_{A}^{3}$: age ≥50.

The results of the ternary classification for each individual sensor are given in Figure 5. Here, the classification estimated the assignment to three classes according to three age groups ($C_{A}^{1}$: age <40; $C_{A}^{2}$: 40 ≤ age < 50; $C_{A}^{3}$: age ≥50) of participants. Similar to the previous classification tasks, the classification based on the combined features of accelerations and angular velocities performs better than the individual ones. More precisely, age classification results for the combined features are (listed by sensor in descending order of rates): lower back (88.822%), chest (88.818%), left ankle (85.74%), right wrist (83.50%). Table 6 presents 10-fold cross-validation results of age classification, including correct classification accuracy, sensitivity, specificity, the positive predictive value (PPV) of each class and the average PPV of all classes. PPV${}_{C_{1}}$ represents the PPV of the class $C_{A}^{1}$; PPV${}_{C_{2}}$ represents the PPV of the class $C_{A}^{2}$; and PPV${}_{C_{3}}$ represents the PPV of the class $C_{A}^{3}$.

### 3.4. Contribution of Individual Features to Classification Results

The contribution of each of the employed features in all three classification tasks was homogenous in the sense that there is not one outstanding feature with a major contribution to the classification results. In all experiments, we made the following observation: in sum, accelerations contributed more to the overall results than angular velocities. However, the combination of the two feature types did better than accelerations or angular velocities individually. Random forest’s permutation-based variable importance measures have been used to evaluate the contribution of individual features in the overall classification results. For further details, refer to the works of Breimann [29] and Louppe *et al.* [37].

In detail, the classification results related to sensors at different locations can depend on quite different feature sets. In the following, we will give an overview of the most important contributors for each of the locations.

#### 3.4.1. Gender Classification

For the location at the chest, angular velocities (around the y-axis, *i.e.*, transverse axis) contributed most, especially the standard deviation, max, energy, and RMS. These are related to the rotation of the upper body around a horizontal axis over the course of the motion. Note that this is not a contradiction to our other claims. Furthermore, the amplitude of the accelerations along the x-axis, *i.e.*, the cranio-caudal axis, is of high importance. For the lower back, the most important features are associated with acceleration of the z-axis. This corresponds to changes in the velocity of the hip movement within the sagittal plane, *i.e.*, front to back. In addition, angular velocities associated with the z-axis, *i.e.*, rotation around the anteroposterior axis (swinging of hips), contribute significantly to the results. Furthermore, the amplitude of the accelerations along the x-axis, *i.e.*, the cranio-caudal axis, is also of high importance. For the right wrist, features associated with acceleration along the y- and z-axes are top contributors. Particularly, minimum, maximum and entropy acceleration values associated with dorso-ventral, as well as lateral movement of the hand play a more important part in the classification. Furthermore, the RMS and energy of angular velocities associated with the z-axis are important. This is also linked to the swinging of the hand in the lateral direction.

**Figure 6:**
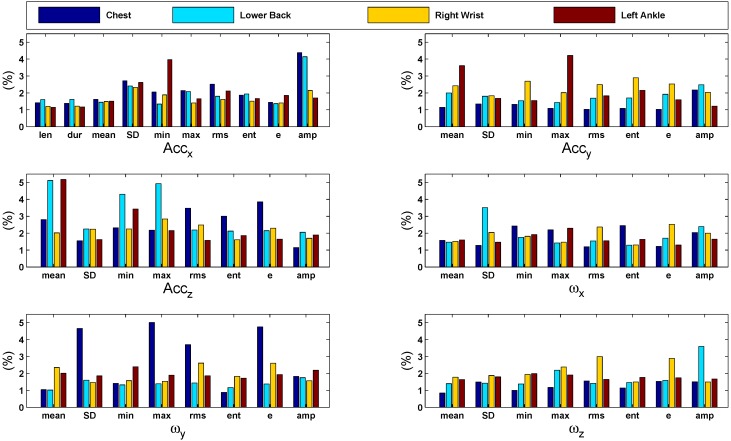
Bar graphs of the features’ importance computed during gender classification using the 10-fold cross-validation strategy. The graphs present a comparison of the importance of each feature (in %) with respect to different sensor positions. In general, all features are significantly contributing in the classification task.

For the ankles, the contribution of accelerations along each axis is generally higher compared to the contribution of other single features. Figure 6 shows bar graphs of the features’ importance computed during gender classification. The graphs present a comparison of the importance of each feature (as percentage) with respect to different sensor positions. In general, all features are significantly contributing in the classification task. An overview of contribution percentages where the most important features are highlighted is given in Table 7.

**Table 7:** Features importance computed during gender classification using 10-fold cross-validation strategy. The top 5 contributing features are highlighted with bold text. All values are the percentage.

		Len	Dur	Mean	SD	Min	Max	RMS	Ent	E	Amp
**Chest**	**A**${}_{x}$	1.42	1.39	1.62	2.72	2.05	2.14	2.51	1.87	1.44	**4.38**
	A${}_{y}$	–	–	1.14	1.35	1.32	1.09	1.03	1.08	1.03	2.17
	A${}_{z}$	–	–	2.80	1.55	2.31	2.18	3.48	3.01	**3.85**	1.15
	G${}_{x}$	–	–	1.58	1.28	2.42	2.20	1.20	2.45	1.22	2.04
	G${}_{y}$	–	–	1.04	**4.67**	1.42	**5.02**	3.70	0.88	**4.75**	1.83
	G${}_{z}$	–	–	0.84	1.49	1.00	1.17	1.56	1.14	1.53	1.50
**Lower Back**	**A**${}_{x}$	1.61	1.62	1.45	2.41	1.34	2.08	1.80	1.94	1.36	**4.14**
	A${}_{y}$	–	–	1.99	1.80	1.53	1.43	1.69	1.70	1.92	2.48
	A${}_{z}$	–	–	**5.11**	2.25	**4.30**	**4.93**	2.19	2.12	2.15	2.05
	G${}_{x}$	–	–	1.47	3.51	1.75	1.42	1.54	1.29	1.71	2.39
	G${}_{y}$	–	–	1.02	1.60	1.33	1.39	1.44	1.16	1.38	1.75
	G${}_{z}$	–	–	1.40	1.42	1.38	2.20	1.42	1.46	1.59	**3.60**
**Right Wrist**	**A**${}_{x}$	1.20	1.21	1.49	2.33	1.88	1.41	1.62	1.52	1.40	2.14
	A${}_{y}$	–	–	2.43	1.83	**2.69**	2.01	2.49	**2.89**	2.53	2.04
	A${}_{z}$	–	–	2.02	2.24	2.25	**2.84**	2.49	1.62	2.30	1.70
	G${}_{x}$	–	–	1.52	2.04	1.82	1.47	2.36	1.31	2.52	2.00
	G${}_{y}$	–	–	2.35	1.46	1.57	1.53	2.61	1.83	2.61	1.57
	G${}_{z}$	–	–	1.78	1.88	1.94	2.38	**3.00**	1.49	**2.89**	1.50
**Left Ankle**	**A**${}_{x}$	1.15	1.17	1.52	2.62	**3.97**	1.65	2.12	1.66	1.86	1.71
	A${}_{y}$	–	–	**3.61**	1.68	1.55	**4.21**	1.83	2.15	1.59	1.22
	A${}_{z}$	–	–	**5.17**	1.62	**3.43**	2.15	1.58	1.86	1.65	1.89
	G${}_{x}$	–	–	1.60	1.46	1.92	2.29	1.55	1.64	1.30	1.65
	G${}_{y}$	–	–	2.01	1.86	2.39	1.90	1.86	1.72	1.94	2.19
	G${}_{z}$	–	–	1.64	1.80	1.99	1.90	1.65	1.77	1.75	1.67

#### 3.4.2. Body Height Classification

For the location at the chest, accelerations along the z-axis contributed most, especially the mean, minimum, maximum and energy. These are associated with the motion of the upper body in the dorso-ventral direction. Furthermore, the minimum accelerations along the x-axis, *i.e.*, the cranio-caudal axis, are of importance.

**Figure 7:**
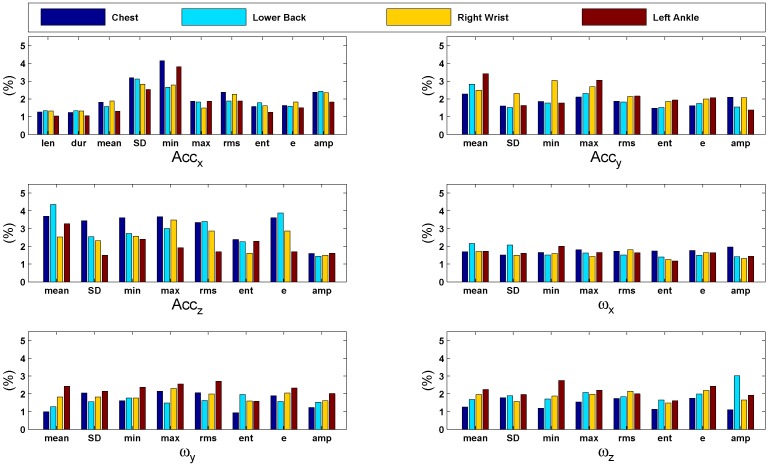
Bar graphs of the features’ importance computed during body height classification using the 10-fold cross-validation strategy. The graphs present a comparison of the importance of each feature (in %) with respect to different sensor positions. In general, all features are significantly contributing in the classification task.

For the lower back, the most important features are associated with acceleration of the z-axis, especially the mean, maximum, RMS and energy. This corresponds to changes in the velocity of the movement of the hips within the sagittal plane, *i.e.*, front to back. In addition, the minimum of the accelerations in the x-axis contributes significantly to the results. These are linked to the movement of the hips along the cranio-caudal axis (up and down). For the right wrist, features associated with acceleration along each of the three axes contribute significantly. Particularly, maximum, RMS and energy values associated with dorso-ventral movement of the hand play a more important part. For the ankles, also the contribution of accelerations along each axis is generally high. Additionally, angular velocities associated with the rotation of the feet from side to side (around the z-axis) are significant contributors. Figure 7 shows bar graphs of the feature contribution computed during body height classification. The graphs present a comparison of the importance of each feature (as percentage) with respect to different sensor positions. In general, all features are significantly contributing in the classification task. An overview of the contribution percentages where the most important features are highlighted is given in Table 8.

**Table 8:** Features’ importance computed during body height classification using the 10-fold cross-validation strategy. The top 5 contributing features are highlighted with bold text. All values are the percentage.

		Len	Dur	Mean	SD	Min	Max	RMS	Ent	E	Amp
**Chest**	**A**${}_{x}$	1.27	1.24	1.81	3.19	**4.15**	1.87	2.38	1.57	1.63	2.38
	A${}_{y}$	–	–	2.28	1.61	1.86	2.10	1.87	1.48	1.62	2.09
	A${}_{z}$	–	–	**3.69**	3.44	**3.62**	**3.67**	3.34	2.38	**3.60**	1.60
	G${}_{x}$	–	–	1.70	1.52	1.65	1.81	1.72	1.74	1.77	1.96
	G${}_{y}$	–	–	1.00	2.05	1.61	2.15	2.06	0.94	1.89	1.22
	G${}_{z}$	–	–	1.25	1.77	1.19	1.54	1.74	1.13	1.75	1.11
**Lower Back**	**A**${}_{x}$	1.34	1.33	1.57	**3.12**	2.65	1.82	1.89	1.78	1.59	2.43
	A${}_{y}$	–	–	2.83	1.51	1.77	2.31	1.82	1.51	1.73	1.54
	A${}_{z}$	–	–	**4.35**	2.54	2.73	**3.01**	**3.40**	2.26	**3.88**	1.44
	G${}_{x}$	–	–	2.16	2.08	1.52	1.63	1.52	1.40	1.50	1.42
	G${}_{y}$	–	–	1.28	1.55	1.76	1.49	1.62	1.96	1.57	1.53
	G${}_{z}$	–	–	1.69	1.89	1.71	2.08	1.83	1.65	1.99	3.02
**Right Wrist**	**A**${}_{x}$	1.33	1.33	1.89	**2.83**	2.78	1.49	2.26	1.62	1.82	2.35
	A${}_{y}$	–	–	2.48	2.31	**3.03**	2.68	2.14	1.86	1.99	2.07
	A${}_{z}$	–	–	2.53	2.31	2.56	**3.48**	**2.87**	1.62	**2.86**	1.50
	G${}_{x}$	–	–	1.71	1.49	1.60	1.43	1.81	1.26	1.64	1.32
	G${}_{y}$	–	–	1.82	1.82	1.76	2.30	1.98	1.59	2.04	1.61
	G${}_{z}$	–	–	1.95	1.57	1.87	1.97	2.14	1.48	2.19	1.65
**Left Ankle**	**A**${}_{x}$	1.04	1.06	1.31	2.53	**3.81**	1.86	1.89	1.26	1.50	1.82
	A${}_{y}$	–	–	**3.41**	1.62	1.77	**3.06**	2.16	1.94	2.06	1.38
	A${}_{z}$	–	–	**3.28**	1.51	2.40	1.92	1.69	2.28	1.70	1.61
	G${}_{x}$	–	–	1.73	1.61	2.00	1.65	1.65	1.18	1.65	1.44
	G${}_{y}$	–	–	2.42	2.14	2.37	2.56	2.71	1.57	2.33	2.01
	G${}_{z}$	–	–	2.24	1.95	**2.75**	2.21	2.00	1.61	2.43	1.92

#### 3.4.3. Age Classification

**Figure 8:**
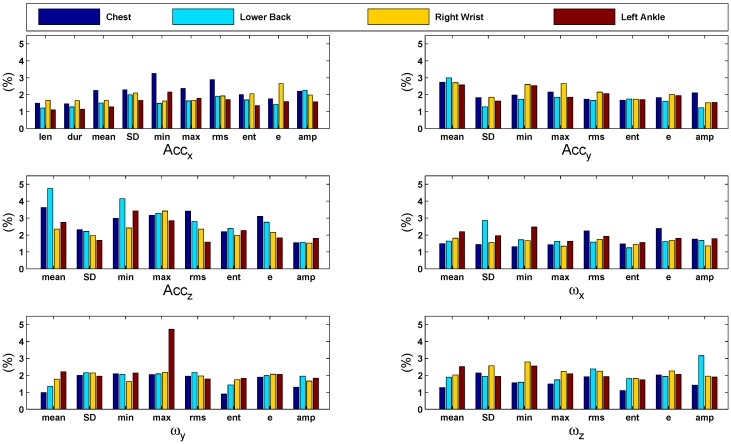
Bar graphs of the features’ importance computed during age classification using the 10-fold cross-validation strategy. The graphs present a comparison of the importance of each feature (in %) with respect to different sensor positions. In general, all features are significantly contributing in the classification task.

For the location at the chest, the importance of the features is similarly distributed as in the height classification results: accelerations along the z-axis contributed most, especially the mean, maximum, RMS and energy. These are associated with the motion of the upper body in the dorso-ventral direction. Furthermore, the minimum acceleration along the x-axis, *i.e.*, the cranio-caudal axis, is important. For the lower back, the most important features are associated especially with acceleration of the z-axis. This is similar to the results found in the height classification scenario and corresponds to changes in the velocity of the movement of the hips within the sagittal plane, *i.e.*, front to back. For the right wrist, features associated with acceleration along each of the three axes contribute significantly. Additionally, the minimum angular velocity associated with rotation around the z-axis, *i.e.*, swinging laterally, is important. For the ankles, the contribution of features associated with lateral acceleration is high. Additionally, angular velocities associated with swinging of the feet from side to side (around the z-axis), as well as rolling over from heel to toes (rotation around the y-axis) are significant contributors. Figure 8 shows bar graphs of the features’ importance computed during age classification. The graphs present a comparison of the importance of each feature (as percentage) with respect to different sensor positions. In general, all features are significantly contributing in the classification task. An overview of contribution percentages where the most important features are highlighted is given in Table 9.

**Table 9:** Features’ importance computed during age classification using the 10-fold cross-validation strategy. The top 5 contributing features are highlighted with bold text. All values are the percentage.

		Len	Dur	Mean	SD	Min	Max	RMS	Ent	E	Amp
**Chest**	**A**${}_{x}$	1.50	1.46	2.26	2.29	**3.25**	2.37	2.89	2.00	1.75	2.21
	A${}_{y}$	–	–	2.74	1.83	1.97	2.16	1.73	1.67	1.83	2.11
	A${}_{z}$	–	–	**3.63**	2.31	2.98	**3.17**	**3.40**	2.19	**3.11**	1.54
	G${}_{x}$	–	–	1.49	1.44	1.31	1.43	2.24	1.47	2.38	1.76
	G${}_{y}$	–	–	0.99	2.00	2.09	2.05	1.95	0.90	1.90	1.30
	G${}_{z}$	–	–	1.28	2.14	1.56	1.49	1.92	1.10	2.02	1.42
**Lower Back**	**A**${}_{x}$	1.22	1.28	1.51	1.99	1.49	1.63	1.91	1.69	1.42	2.26
	A${}_{y}$	–	–	**2.99**	1.29	1.73	1.85	1.65	1.74	1.61	1.22
	A${}_{z}$	–	–	**4.75**	2.22	**4.15**	**3.28**	2.80	2.38	2.76	1.56
	G${}_{x}$	–	–	1.64	2.86	1.72	1.63	1.58	1.25	1.62	1.67
	G${}_{y}$	–	–	1.35	2.16	2.06	2.09	2.17	1.44	1.98	1.95
	G${}_{z}$	–	–	1.90	1.94	1.59	1.74	2.38	1.81	1.94	**3.17**
**Right Wrist**	**A**${}_{x}$	1.68	1.65	1.66	2.10	1.62	1.66	1.93	2.05	**2.65**	1.98
	A${}_{y}$	–	–	**2.71**	1.85	2.60	**2.65**	2.15	1.73	2.00	1.52
	A${}_{z}$	–	–	2.35	1.96	2.42	**3.42**	2.35	1.97	2.17	1.52
	G${}_{x}$	–	–	1.81	1.56	1.67	1.34	1.75	1.44	1.68	1.35
	G${}_{y}$	–	–	1.78	2.14	1.63	2.18	1.97	1.74	2.07	1.67
	G${}_{z}$	–	–	2.03	2.57	**2.79**	2.23	2.25	1.81	2.26	1.95
**Left Ankle**	**A**${}_{x}$	1.10	1.15	1.29	1.66	2.16	1.79	1.70	1.35	1.59	1.58
	A${}_{y}$	–	–	**2.58**	1.63	2.54	1.84	2.06	1.70	1.94	1.54
	A${}_{z}$	–	–	**2.75**	1.68	**3.42**	**2.84**	1.58	2.27	1.83	1.80
	G${}_{x}$	–	–	2.20	1.96	2.47	1.62	1.93	1.56	1.81	1.78
	G${}_{y}$	–	–	2.21	1.96	2.14	**4.72**	1.79	1.81	2.06	1.84
	G${}_{z}$	–	–	2.52	1.94	2.56	2.09	1.93	1.74	2.06	1.90

### 3.5. Classification Results Based on Restriction to Subgroups

Since the correlation between body height and gender is very high (on average, men are taller than women), we performed a gait-based classification task on each of the groups of female and male participants in order to present height classification results that are independent of this particular phenomenon. Moreover, we also performed age classification on the data of each subgroup (female *vs.* male) separately. The number of subjects present in the study did not allow for ternary classification of subgroups (see Table 5 for the population characteristics). Therefore, there were two different classes in the height-related experiment: $C_{H}^{1}$ = the body height of the subject is less than or equal to $t_{h}$ cm; $C_{H}^{2}$ = the body height of the subject is greater than $t_{h}$ cm ($t_{h}=180$ for male, $t_{h}=170$ for female subjects). In the age-related experiment, assigned classes were: $C_{A}^{1}$ = the subject is less than or equal to $t_{a}$ years old; $C_{A}^{2}$ = the subject is greater than $t_{a}$ years old ($t_{a}=40$ for male, $t_{a}=50$ for female subjects).

Table 10 shows an overview of the results. It is quite clear that the results are very good in all cases with the classification rate higher than 90% in all but two cases (89.34% and 87.97% for the right wrist sensor in both female groups). The results also present balanced sensitivity, specificity, the positive predictive value (PPV) of each class and the average PPV of all classes. For body height classification, PPV${}_{C_{1}}$ represents the PPV of the class $C_{H}^{1}$, and PPV${}_{C_{2}}$ represents the PPV of the class $C_{H}^{2}$. For age classification, PPV${}_{C_{1}}$ shows the PPV of the class $C_{A}^{1}$, and PPV${}_{C_{2}}$ shows the PPV of the class $C_{A}^{2}$.

**Table 10:** Results of body height and age classifications within participant subgroups using 10-fold cross-validation. The results show balanced correct classification rates, sensitivity, specificity, the positive predictive value (PPV) of each class and the average PPV of all classes.

Classification Task	Body Part	Sensor	Class. Rate	Sens.	Spec.	PPV${}_{{\text{C}}_{1}}$	PPV${}_{{\text{C}}_{2}}$	Avg. PPV
**Body Height Classification**								
**Male Group**	Chest	A${}_{xyz}$, G${}_{xyz}$	95.06	96.74	92.72	94.87	95.33	95.10
	Lower Back	A${}_{xyz}$, G${}_{xyz}$	93.46	94.82	91.61	93.93	92.81	93.37
	Right Wrist	A${}_{xyz}$, G${}_{xyz}$	93.50	96.77	89.07	92.31	95.32	93.81
	Left Ankle	A${}_{xyz}$, G${}_{xyz}$	93.27	94.91	91.20	93.16	93.41	93.29
**Female Group**	Chest	A${}_{xyz}$, G${}_{xyz}$	91.18	92.84	89.07	91.49	90.77	91.13
	Lower Back	A${}_{xyz}$, G${}_{xyz}$	93.22	96.06	89.63	92.13	94.73	93.43
	Right Wrist	A${}_{xyz}$, G${}_{xyz}$	89.34	92.97	84.90	88.30	90.78	89.54
	Left Ankle	A${}_{xyz}$, G${}_{xyz}$	92.71	94.71	90.08	92.59	92.86	92.73
**Age Classification**								
**Male Group**	Chest	A${}_{xyz}$, G${}_{xyz}$	93.36	93.12	93.60	93.90	92.79	93.34
	Lower Back	A${}_{xyz}$, G${}_{xyz}$	93.61	93.45	93.77	94.01	93.19	93.60
	Right Wrist	A${}_{xyz}$, G${}_{xyz}$	93.55	94.40	92.65	93.19	93.95	93.57
	Left Ankle	A${}_{xyz}$, G${}_{xyz}$	92.65	92.69	92.62	92.58	92.73	92.65
**Female Group**	Chest	A${}_{xyz}$, G${}_{xyz}$	92.78	90.04	95.29	94.59	91.27	92.93
	Lower Back	A${}_{xyz}$, G${}_{xyz}$	95.05	95.78	94.39	93.92	96.11	95.01
	Right Wrist	A${}_{xyz}$, G${}_{xyz}$	87.97	88.79	87.20	86.62	89.29	87.96
	Left Ankle	A${}_{xyz}$, G${}_{xyz}$	90.80	87.37	93.74	92.29	89.64	90.96

### 3.6. Subject-Wise Cross-Validation

**Figure 9:**
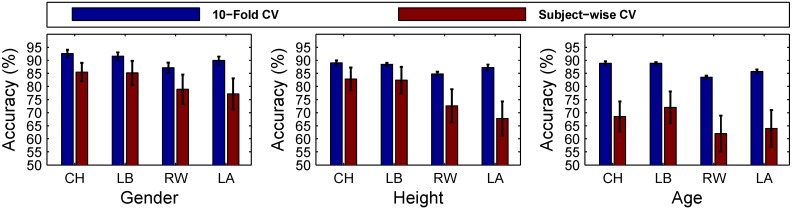
A comparison of correct classification accuracy of group classification tasks (gender, height and age) using 10-fold cross-validation and subject-wise cross-validation. Sensor positions include: chest (CH), lower back (LB), right wrist (RW) and left ankle (LA). The 10-fold cross-validation model outperforms the subject-wise cross-validation model in all cases.

In order to show that our results are not caused by over-fitting the classification to specific subjects rather than learning the properties, we are looking for (gender, height, age), a subject-wise cross-validation model was also employed (as explained in Section 2.8). Table 11 presents the classification results of subject-wise cross-validation for all three group classification tasks: gender, height and age. The feature set contained all features of 6D accelerations and angular velocities (50 in total). For each sensor position, sensitivity, specificity, the PPV of each class and the average PPV of all classes were also computed. A comparison of the classification results of group classification tasks using 10-fold cross-validation and subject-wise cross-validation for chest (CH), lower back (LB), right wrist (RW) and left ankle (LA) is presented in Figure 9. It is clearly observable that 10-fold cross-validation outperforms subject-wise cross-validation in all cases.

**Table 11:** Subject-wise classification results of different classification categories: gender, height and age. The results show balanced correct classification rates, sensitivity, specificity, the positive predictive value (PPV) of each class and the average PPV of all classes.

Classification Task	Body Part	Sensor	Class. Rate	Sens.	Spec.	PPV${}_{C_{1}}$	PPV${}_{C_{2}}$	PPV${}_{C_{3}}$	Avg. PPV
**Gender Classification**	Chest	A${}_{xyz}$, G${}_{xyz}$	85.48	85.09	85.88	86.28	84.66	–	85.47
	Lower Back	A${}_{xyz}$, G${}_{xyz}$	87.95	85.71	89.71	86.74	88.88	–	87.81
	Right Wrist	A${}_{xyz}$, G${}_{xyz}$	78.90	73.50	82.69	74.89	81.63	–	78.26
	Left Ankle	A${}_{xyz}$, G${}_{xyz}$	77.14	82.32	72.67	72.17	82.68	–	77.43
**Body Height Classification**	Chest	A${}_{xyz}$, G${}_{xyz}$	82.87	79.13	91.23	75.00	71.20	91.81	79.34
	Lower Back	A${}_{xyz}$, G${}_{xyz}$	84.38	84.88	92.02	83.18	81.98	87.23	84.13
	Right Wrist	A${}_{xyz}$, G${}_{xyz}$	72.61	71.98	86.31	80.02	58.66	79.10	72.59
	Left Ankle	A${}_{xyz}$, G${}_{xyz}$	67.78	67.84	83.92	84.96	59.57	61.60	68.71
**Age Classification**	Chest	A${}_{xyz}$, G${}_{xyz}$	68.54	69.38	84.47	59.79	85.62	70.28	71.90
	Lower Back	A${}_{xyz}$, G${}_{xyz}$	72.00	72.05	85.61	63.95	72.28	85.23	73.82
	Right Wrist	A${}_{xyz}$, G${}_{xyz}$	61.99	61.72	80.96	53.01	68.50	62.84	61.45
	Left Ankle	A${}_{xyz}$, G${}_{xyz}$	63.95	63.31	81.91	60.59	55.31	72.88	62.93

In the case of gender classification using chest and lower back sensors, the classification rates are 7.08% and 6.37% lower than 10-fold cross-validation. For right wrist and left ankle sensors, the classification rates are 8.26% and 12.83% lower than 10-fold cross-validation. In the case of height classification using chest and lower back sensors, the classification rates are 6.18% and 6.07% lower than 10-fold cross-validation. For right wrist and left ankle sensors, the classification rates are 12.18% and 19.50% lower than 10-fold cross-validation.

For the age classification task, a sharp decline in the classification rates is observable in subject-wise cross-validation. For chest and lower back sensors, the classification rates are 20.28% and 16.82% lower than 10-fold cross-validation. For right wrist and left ankle, the classification rates are 21.51% and 21.79% lower than 10-fold cross-validation. The main reason for such a sharp decline is because of the unbalanced population in classes $C_{A}^{1}$, $C_{A}^{2}$ and $C_{A}^{3}$ with a subject ratio of 9:6:11.

On the level of subject-wise cross-validation, it is also possible to address the questions of the invariance of the features within the different steps of a walking sequence or to come up with random forest regressions for age and height. Not surprisingly, almost all steps of one walking sequence were classified identically; 99.1% for gender classification, 98.7% for height classification and 98.4% for age classification. When performing a random forest regression instead of a classification, we obtained age classifications with an average RMS error of about 11.51 years and height classification with an average RMS error of about 9.14 cm.

## 4. Discussion

### 4.1. Summary of Findings

The general problem we tackled is the estimation of soft biometric information from one single step recorded by one inertial sensor. We did so by solving different classification tasks based on the motion data of human walking steps represented by accelerations and angular velocities. Data were recorded by one sensor placed at various locations on the human body, namely the chest, the lower back, the wrist and the ankle. The results show that these classification tasks can be solved well by using accelerometers and/or gyroscopes at any of the given locations. The classification rates were highest for sensors located at the lower back and chest in each of the experiments, but still convincingly high when the sensor is attached to the wrist or ankle.

Our analysis of the feature sets used in each of the experiments has made clear that there is not one feature mainly responsible for any of the distinctions necessary for a classification. However, the feature importance in each of the classifications gave pointers as to what combination of features produces the best results. The most important findings were that angular velocities did not perform better than accelerations.

### 4.2. Comparison with Existing Research

It is not surprising that information about the gender can be recovered by analysis of chest or lower back movement. The effects of marker placement and viewpoint selection for recording locomotion are discussed extensively in the works of Troje [2], as was the high relevance of hip movement for gender classification by human observers. However, we have presented new findings, namely that accelerations associated with wrist and ankle movement alone allow for classification of gender, as well. To our knowledge, we are also the first to show that classification of height and age groups is possible from non-visual features. This is as yet done by solely relying on image- or video-based features. Makihara *et al.* [38] introduce a paper on gait-based age estimation by Gaussian process regression on silhouette-based features of bodies (contrary to face-based age estimation, as presented by Stewart *et al.* [39]). Their investigation was based on standard resolution video data. They have constructed a whole-generation database of over 1000 individuals, their age ranging from two to 94.

Our initial situation is clearly different from this in terms of sensor modalities. The use of commercial smart phones and wearables is an attractive chance to monitor biometric properties nowadays. Mobile phones and smart devices are a convenient platform for recording information in an every-day setup. Our experiments have shown that information recorded by a single sensor, such as a smart device, suffices for the estimation of basic soft biometric properties. Particularly, the wrist was an important subject for tests, because smart devices are commonly worn at that location.

Estimating biometric properties based on motion data makes sense in a number of different scenarios. In some of them, the focus may be on hard biometric properties in order to facilitate online identity checks and close security gaps. A number of previous works have shown that identification and authentication problems can be solved by classification of motion data acquired by mobile devices. Derawi and Bours [40] show that recognition of specific users can be done in real-time based on data collected by mobile phones. Their method can correctly identify enrolled users based on learning templates of different walking trials.

On the other hand, attention may be directed to soft biometric properties. Monitoring health or preventing and curing injury are use cases that represent this idea. Previous works have shown that accelerometers are well suited for detection and recognition of events and activity. In their paper on sensory motor performance, Albert *et al.* [41] discuss a new method to classify different types of falls in order to rapidly assess the cause and necessary emergency response. They present very good results classifying accelerometer data acquired by commercial mobile phones, which were attached to the lower backs of test subjects. In their comparative evaluation of five machine learning classifiers, support vector machines performed best, achieving accuracy values near 98%. Classification by decision trees only performed second best in their experiments at 94% to 98% accuracy for fall detection and at 98% to 99% accuracy for fall type classification. In their paper on gait pattern classification, Von Tscharner *et al.* [42] even conclude that a combination of PCA, SVM and ICA is most reliable dealing with high intra- and inter-subject variability. However, in their survey on mobile gait classification, Schneider *et al.* [43] make an attempt to settle the disagreement about suitable classification algorithms. In their study, they conclude that random forest is best suited for the classification of gait-related properties. In our setup, we decided to use random forest in order to produce comparable results. One additional benefit of this choice is that there is a low number of parameters that have to be chosen. Furthermore, the random forest method enables computing the significance and importance of each feature in overall classification. This helped us to investigate and perform a comparative study of the features’ importance for each sensor position in different classification tasks.

### 4.3. Limitations

Since our database is much smaller than the one introduced by Makihara *et al.* [38] and the variety of biometric features was also smaller (e.g., age covered only three decades), our experiments can only serve as proof of concept for now. Testing classifiers of non-image-based features on a larger database comprising wider ranges of biometric properties is a direction for future work.

Another limitation of our database is that it only consists of data belonging to patients with complaints of back pain. It will be worthy to perform further experiments to record data of participants without back pain (control group). Classification tasks can then be performed for the patient group, the control group and a combination of both.

One noteworthy limitation we had to face in our experiments is a possible uncertainty of sensor placement. Irrespective of how carefully each involved sensor is placed, the accuracy of placement depends on physical characteristics of test subjects, which may vary between individuals to some extent.

## 5. Conclusions and Future Work

We have classified biometric information based on the data of a single inertial-measurement unit collected on a single step. As a novel empirical finding, we have shown that single steps of normal walking already reveal biometric information about gender, height and age quite well, not only for measurements of lower back movements or chest movements, but also for wrist movements or ankle movements. Using standard 10-fold cross-validation, the classification rates have been for gender classification: 87.16% (right wrist sensor) to 92.57% (chest sensor); height classification: 84.78% (right wrist sensor) to 89.05% (chest sensor); age classification: 83.50% (right wrist sensor) to 88.82% (chest, lower back sensor). When using the rather strict subject-wise evaluations, the classification rates are somewhat lower for gender by 6.37% (lower back sensor) to 12.83% (left ankle) compared to the results of 10-fold cross-validation. For height classification, the classification rates using subject-wise evaluation are 6.07% (lower back sensor) to 19.50% (left ankle sensor) lower, and for age classification, 16.82% (lower back sensor) to 21.79% (left ankle sensor). These values can be seen as “lower bounds” on the possible classification rates on the biological variations, since also our feature selection, as well as our used machine learning techniques might not be optimal. Especially, a good estimate of the direction of gravity should improve the results; at sensors position with less change in orientation (chest, lower back), the classification rates had been better than at the ones with higher change (wrist, ankle). In future work, we will try to adopt a model-based estimate of body-part orientation using techniques similar to the ones used in [17] to come up with such estimates.

On the side of the basic science questions about human movement control, we want to address questions about to which degree the movement patterns can be “spoofed” by trained and untrained persons in future work. We will perform tests asking probands to try to walk like the other gender, to pretend to have another age or to have another height, *etc*.

On the technological side, our work should help to gain information on the user by smartwatches, smartphones or smart shoes, given the fact that many sensor systems for consumer electronics are limited: long time recordings can be done in low frame rates only or high speed measurements can be done for a limited amount of time, to save battery life time. Thus, it is more and more important to get information out of sparse sensor readings. Our work presents a technique where biometric parameters can be estimated from single steps. These biometric parameters can be used for further analysis of motions that are recorded with lower frame rates. Compared to previous work, where full sequences are considered for classification, we see this as a strong improvement.

However, our work also demonstrates the sensitivity of sensor data of such devices with respect to privacy concerns: already, the information on a single step recorded from a smartphone or smartwatch reveals personal information on gender, height and age.
